# Pharmacokinetic Studies of Baclofen Are Not Sufficient to Establish an Optimized Dosage for Management of Alcohol Disorder

**DOI:** 10.3389/fpsyt.2018.00485

**Published:** 2018-10-05

**Authors:** Nicolas Simon, Nicolas Franchitto, Benjamin Rolland

**Affiliations:** ^1^Aix Marseille Univ, INSERM, IRD, SESSTIM, Hop Sainte Marguerite, Service de Pharmacologie Clinique, CAP, Marseille, France; ^2^Service d'addictologie, Centre Hospitalier Universitaire de Toulouse, Institut National de la Santé et de la Recherche Médicale (INSERM) UMR 1027, Université Paul Sabatier, Toulouse, France; ^3^Service Universitaire d'Addictologie, Pôle UP-MOPHA, CH Le Vinatier, Bron, France; ^4^Univ Lyon, Inserm U1028, CNRS UMR5292, UCBL, CRNL, Bron, France

**Keywords:** clinical pharmacokinetics, baclofen, alcohol use disorder, modeling, GABA

## Abstract

Several clinical randomized trials have evaluated the interest of baclofen in patients with alcohol use disorder. Depending on the study design and the inclusion criteria, the results vary from enthusiastic to pessimistic. However, all researchers and practitioners agree that they observe a wide variability in the therapeutic responses. If some patients exhibit a clinical response at low doses, ~40 mg daily, others require doses higher than 300 mg. Before multiplying new other clinical trials, it is required to better understand the reason of this variability. Several mechanisms may be responsible for providing different effects with an identical daily dose. Especially, each pharmacokinetic step, absorption, distribution, metabolism, and elimination may lead to a different exposure after an identical dose. Absorption may imply a saturation process limiting the bioavailability (F) of baclofen in some patients. In such a situation, food, or drug-drug interaction can change the absorption rate of the drug modifying the maximum concentration (Cmax) and area under the curve (AUC). Distribution and brain penetration across the blood-brain barrier may depend of a specific transporter. These transporters are subject to genetic polymorphism and drug-drug interaction. Finally, elimination may be increased by a specific secretion pathway. This review describes all available pharmacokinetic data on these different pharmacokinetics steps aiming to identify the source of variability of baclofen in patients with alcohol use disorder.

## Introduction

Baclofen is a racemic drug with GABA-B receptor agonist properties. It is widely prescribed as a spasmolytic agent to treat spasticity caused by central nervous system lesions or dysfunction (1). For several years baclofen has been prescribed off-label in Alcohol Use Disorder (AUD) patients to prevent relapse or to reduce drinking (2). This new indication is even authorized in France as “temporary recommendation for use.” Unfortunately, the use of this compound is made difficult by the lack of knowledge on prescription guidelines, dosage and characteristics of patients with the highest probability of a clinical response (2, 3). In clinical practice, a wide inter-individual variability of dose required to obtain an effect is often described from 30 up to 300 mg per day (2). Curiously, randomized clinical trials have been performed before full evaluation of pharmacokinetic properties and even a clear pharmacodynamic proof of concept. This lack of a standard clinical drug development in this new indication may explain the discrepancy between clinical trials (4–7). Thus, the aim of this review is to recapitulate the pharmacokinetic properties already reported and to identify the studies required to optimize its evaluation in this new indication.

## Absorption

Only oral formulation with an immediate release is currently available. In healthy volunteers the time to reach the peak concentration in plasma after administration (Tmax) varied between 1 h (8) and 2.79 h (9). Other pharmacokinetic studies in healthy volunteers found similar results: 1.13 h (10), 1.2 h (11), or 2 h (12, 13). The pharmacokinetic studies performed in AUD patients have been realized in real life conditions. Such studies used sparse sampling instead of full profiles, which did not allow a direct observation of Tmax. Meanwhile a formula (Equation 1) can be used for computing Tmax from the constant of absorption (Ka) and the constant of elimination (Ke). The calculated Tmax obtained from two population pharmacokinetics studies are 1.23 h (14) and 1.74 h (3).

(1) $$\begin{array}{l} Tmax=log\left(Ka/Ke\right)/\left(Ka-Ke\right) \end{array}$$

The baclofen's Tmax indicates a rapid absorption of the compound from the intestinal tract to the bloodstream and should not be confused with the time required to observe an effect. Indeed, some effects may change over time in parallel with plasma concentrations, but this has never been described for baclofen. Unless a direct relationship is described between plasma concentration and a baclofen effect, the Tmax indicates nothing more than a laboratory value. This rapid absorption can be explained by the site of penetration into the digestive tract wall. The upper small intestine completely absorbs baclofen with almost no contribution of the colon (15). This characteristic suggests that baclofen absorption could be modified when prescribed in patients who have undergone a bariatric surgery. Unfortunately, no pharmacokinetic data are currently available in such situation and any prescription to these patients should be made with caution.

Several mechanisms are suggested to explain the ability of a compound to pass through the digestive barrier and to enter into the bloodstream. Depending on its chemical and physical properties, a compound will be absorbed by a passive process, a transporter or both of them. *In vitro* and *in vivo* studies have been performed in different species to understand the mechanism of baclofen absorption. Even if more studies are required to fully understand the compound behavior in the digestive wall, some convincing results described that baclofen used more than one intestinal carrier system (16). At least baclofen shares a saturable transport mechanism with essential branched-chain aminoacid (BCAA) such as leucine (17, 18). Since aminoacids are a normal component of food, a competitive inhibition could arise. However, a study performed with healthy volunteers under fasting condition vs. a standard meal did not find a significantly influence of food neither on the rate nor on the extent of baclofen absorption (19).

The absolute oral bioavailability has not been evaluated in AUD patients, but in healthy volunteers it was ~74% for 10 mg (8) and 108, 85.8, and 81.2% for 10, 15, and 20 mg, respectively (10). To summarize, following an oral administration the absorption did not seem to be a limiting step for baclofen.

## Distribution

Baclofen is 30% protein bound into the plasma which suggests that protein-binding changes will not be clinically relevant for routine practice (20). In special circumstances such as a baclofen intoxication, where a hemodialysis could be proposed, the protein binding may have an influence.

The mechanism of baclofen in AUD is suggested to be related with its ability to bind to the GABA-B receptor. So, the concern about baclofen distribution in AUD patients is the rate and extent of penetration into the CNS. With a logD of −0.96 the lipophilicity of baclofen is insufficient to allow a brain penetration by passive diffusion (21). The baclofen brain entry across the blood-brain-barrier is due to an interaction with the large neutral aminoacid transporter (22, 23). However, the resulting influx clearance is counterbalanced by an efficient efflux from the brain through a probenecid-sensitive organic anion transporter 3, OAT3 (SLC22A8) (24, 25). The overall restricted distribution of baclofen in the brain is then the result of an asymmetric transport at the blood-brain-barrier with an efflux transport rate 40-fold higher than the influx transport rate (25). The fact that baclofen is a substrate of OAT3 remains to be confirmed but recent results corroborate its low apparent permeability coefficient (Caco-2 *P*_app_: 0.9 ± 0.7 10^−6^ cm/s) and its unbound brain/blood ratio (26).

**Figure 1:**
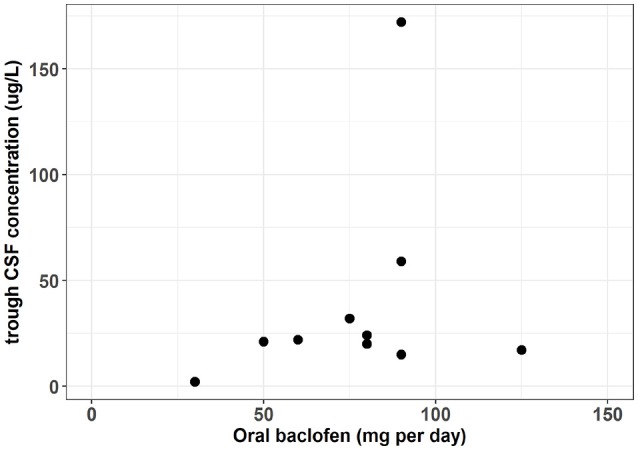
Oral dose vs. trough CSF baclofen concentration in patient with severe spasticity. [From data published by Heetla et al. (1) and individuals CSF trough concentration values kindly communicated by the authors].

In patients with spastic paresis, a study was designed to elucidate whether the therapeutic responses and the side effects were related to plasma concentrations and CSF levels (27). Four hours after the administration, the authors found CSF levels nine to ten times lower than the plasma concentrations. However, optimal therapeutic responses were obtained at very different levels of plasma and CSF baclofen concentrations and without a clear relationship. The analytical method used for CSF estimation was a limitation resulting in numerous samples below the quantification limit and wasn't able to fully describe the interindividual variation. A recent study used a more efficient quantification analysis to describe the baclofen concentrations in CSF (1). The population wasn't AUD patients but patients with various etiology of severe spasticity. Meanwhile interesting information can be extrapolated to baclofen used in AUD. Before the intra-thecal administration, ten patients were treated with a repeated oral dose of baclofen ranging from 30 to 125 mg per day. The resulting individual trough CSF concentrations were available but not showed in the original article. However, the authors kindly agreed to communicate them allowing the description of the relationship between oral doses and CSF trough concentrations (Figure 1). This figure shows that between a dose of 30–125 mg per day the corresponding CSF concentrations seem to vary slightly around 25 μg/L. This result suggests that plasma concentration is probably not a good biomarker of baclofen brain exposure. Interestingly, the patients included in this study were recruited on the basis of a severe spasticity which could not be treated sufficiently with an oral therapy. However, following the intrathecal administration, all these patients achieved an adequate spasmolytic effect. The pharmacokinetic/pharmacodynamic (PK/PD) model of spasticity estimated a CSF concentration at 50% of the maximum effect (EC50) of 194 ug/L (95% CI 112, 350), which is higher than the 25 ug/L obtained with the oral administration. A PK/PD model of baclofen in AUD remains to be identified but the results in patients with spasticity suggest that baclofen brain penetration could be a limiting factor for its efficacy and may explain a large inter-individual variability.

## Metabolism and elimination

A stereoselective metabolic difference between R- and S-baclofen has been recently described (28). In this study no metabolites were observed following an oral administration of the single R-enantiomer. However, the administration of the mixture R- and S- allowed the identification of an oxidative deamination metabolite. This stereoselective metabolism of only the S-enantiomer of baclofen is then followed by a glucuronide conjugation. Meanwhile the overall contribution of metabolism is low with 85% of the dose excreted as unchanged (29). This result may explain that in another study the gamma-hydroxymetabolite was not detected (12).

Baclofen is mainly cleared through kidneys with a fraction of the dose unchanged in the urine ranging from 65% (13) to 80.9% (11). In healthy volunteers a high correlation between the apparent renal clearance of baclofen and the creatinine clearance was described (12). This has been confirmed by a study including four groups of patients with different kidney disease stages (30). The authors found the following significant linear relationship (*R*^2^ = 0.67, *p* < 0.0001) between baclofen clearance (CL/F) and creatinine clearance (CrCL):

(2) $$\begin{array}{l} CL/F\left(L/h\right)=2.25+0.05\times CrCL\left(ml/min\right) \end{array}$$

**Figure 2:**
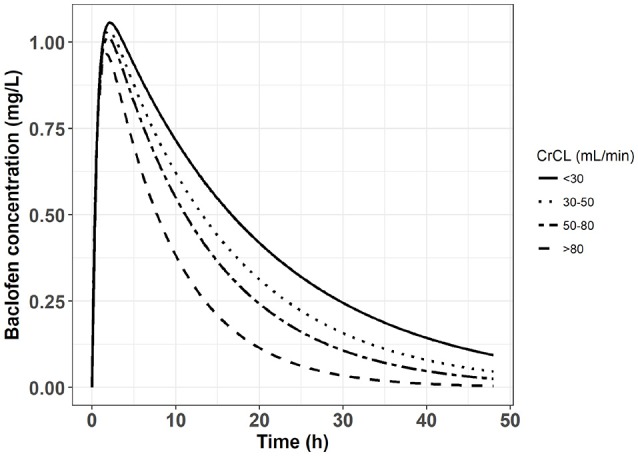
Simulated concentration vs. time profile following an oral dose of 80 mg of baclofen in four groups of patients with different chronic kidney disease stages.

This result suggests that, if baclofen has to be prescribed in patients with chronic kidney disease, a dose reduction should be applied. Using Equation 2 and a one-compartment model of baclofen described by Imbert et al. (3), it was possible to simulate concentration vs. time profiles with different values of baclofen clearance (CL/F) depending on the creatinine clearance. The Figure 2 depicts these baclofen simulated concentrations following an oral dose of 80 mg in patients according to different values of creatinine clearance. Meanwhile none of the pharmacokinetic studies performed in AUD patients identified a significant influence of creatinine clearance on baclofen clearance (3, 14, 31). This surprising result can be explained by the homogeneity of patients included in these studies regarding the renal function.

The baclofen half-life (T1/2) in alcohol-dependent patients with the current immediate release formulation ranged from 3.8 h (14) to 6.4 h (3). These values were obtained for doses up to 180 mg per day (3). It's noticeable that during a baclofen overdose an increase of T1/2 is not always observed (32). In such situation, which may lead to comatose, severe respiratory depression and neurotoxicity, hemodialysis has been effective in reversing baclofen toxicity if the patient had a renal insufficiency (33). A gastro-intestinal decontamination with activated charcoal can also be proposed to avoid a persistent intestinal absorption related to a reduced gastro-intestinal mobility or a pharmacobezoar (34).

To conclude on these pharmacokinetic steps, baclofen exhibited no saturation of elimination with clinical doses used and thus no accumulation. However, in patient with chronic kidney disease a decrease of clearance is expected, and the baclofen dosage should be adjusted accordingly. The guidelines for drug dosing regimens in chronic kidney disease deserve further studies.

## Discussion

Baclofen, as are all drugs, needs to reach its site of action to produce an effect. Because its mechanism of action seems to be related to a binding on the GABA-B receptor in the CNS, the concentrations in plasma are only an indirect marker. It would be more interesting to describe the relationship between an oral dose and CSF concentration, but this kind of study required a catheter located at a spinal level. Other studies such as positron emission tomography imaging could evaluate the baclofen binding to the cerebral GABA-B receptors. For example, we can suggest an imaging study aiming to determine whether patients requiring higher doses and/or patients with poor response correspond to subjects with a lower cerebral binding of baclofen. However, the lack of suitable radiotracers for imaging GABA-B receptors is a current limitation.

Meanwhile a variability in plasma exposure following an oral administration must be evaluated because it is an essential step before the brain penetration. The aim of pharmacokinetic studies is to identify the main source of inter-individual variability such as age, body weight, food, drug-drug interaction, tobacco, renal and hepatic function, genetic polymorphism. However, because the time course of plasma concentration did not necessary follow the time course of an effect, pharmacokinetic studies are not sufficient to establish an optimized dosage. Usually, the increase of plasma concentrations following an administration did not follow the effect onset and likewise the decrease of plasma concentrations presents a different rate than the decrease of the effect. When prescribing baclofen, it's important not to misinterpret Tmax and T1/2: they are not describing the time to effect and the effect disappearance, they are only describing the plasma concentration. Considering the suggested effect of baclofen on craving in AUD, the time to the maximum effect as well as the duration of this effect is still to be described.

Currently only three population pharmacokinetic studies of baclofen have been performed in AUD patients (3, 14, 31). Two studies were based on the same cohort with different objective and population size (3, 31). The population were AUD patients, mainly male (43 males, 24 females) from 29 to 68-year-old, with a body weight ranging from 42 to 128 kg, normal renal function and a daily baclofen dose up to 180 mg (3). The second evaluated population present identical demographic and laboratory characteristics (14). These population pharmacokinetic analysis identified a one-compartment model with first-order input and output to describe the time course of plasma concentrations. However, none of these studies were able to identify factors explaining inter-individual variability.

The pharmacokinetic parameters were not clearly correlated with age, body weight, sex, smoking status, height, aspartate, aminotransferase, alanine aminotransferase, total bilirubine, gamma-glutamyltransferase, and alkaline phosphatases. Both studies found an influence of creatinine clearance on the baclofen clearance, but this relationship never reached a statistical significance (14, 31). Interestingly these studies described a proportional relationship between the oral dose of baclofen and concentrations which did not suggest an accumulation at least up to a daily dose of 180 mg per day.

Concerning putative pharmacokinetic drug-drug interaction, a recent review did not find any of them described in the literature (35). Because baclofen is mainly excreted unchanged in urine, it's unlikely that an inhibition or an induction of its metabolism may have a clinically relevant impact. However, the impact of efflux transporter (OAT3) inhibition in renal tubules and/or in the blood brain barrier deserves an investigation [for a review on OAT family, (36, 37)]. Several compounds such as probenecid and proton pump inhibitors have been described to inhibit OAT3 transport which increases plasma and brain concentrations (38, 39). It could be of interest to combine baclofen with an OAT3 inhibitor to investigate if brain and plasma concentrations are increased and if it is associated with an improvement of the clinical effect.

## Conclusion

Although pharmacokinetic studies already performed in AUD patients described a linear relationship between dose and plasma concentrations, they did not adequately explain the major source(s) of inter-individual variability. Meanwhile caution should be taken if baclofen must be prescribed in patients with chronic kidney disease. A study performed in patients with severe spasticity found no relation between baclofen oral doses and CSF concentrations. This result could partly explain the inter-individual variability of the dose required to reach a clinical effect. Indeed, a concern remains on the ability of baclofen to reach the brain with an appropriate rate and extent and new studies are required to investigate this question.

## Author contributions

NS wrote the first draft of the manuscript. NS, BR, and NF contributed to and have approved the final version of the manuscript.

### Conflict of interest statement

The authors declare that the research was conducted in the absence of any commercial or financial relationships that could be construed as a potential conflict of interest.
